# Formulation and Characterization of Alginate Dialdehyde, Gelatin, and Platelet-Rich Plasma-Based Bioink for Bioprinting Applications

**DOI:** 10.3390/bioengineering7030108

**Published:** 2020-09-09

**Authors:** Lakshmi T. Somasekharan, Naresh Kasoju, Riya Raju, Anugya Bhatt

**Affiliations:** 1 Division of Thrombosis Research, Department of Applied Biology, Biomedical Technology Wing, Sree Chitra Tirunal Institute for Medical Sciences and Technology, Thiruvananthapuram, Kerala 695012, India; s.lakshmit@gmail.com (L.T.S.); riyarajud@gmail.com (R.R.); 2 Division of Tissue Culture, Department of Applied Biology, Biomedical Technology Wing, Sree Chitra Tirunal Institute for Medical Sciences and Technology, Thiruvananthapuram, Kerala 695012, India; naresh.kasoju@sctimst.ac.in

**Keywords:** biofabrication, bioink, hydrogels, growth factor cocktail, bioactive scaffold, printability

## Abstract

Layer-by-layer additive manufacturing process has evolved into three-dimensional (3D) “bio-printing” as a means of constructing cell-laden functional tissue equivalents. The process typically involves the mixing of cells of interest with an appropriate hydrogel, termed as “bioink”, followed by printing and tissue maturation. An ideal bioink should have adequate mechanical, rheological, and biological features of the target tissues. However, native extracellular matrix (ECM) is made of an intricate milieu of soluble and non-soluble extracellular factors, and mimicking such a composition is challenging. To this end, here we report the formulation of a multi-component bioink composed of gelatin and alginate -based scaffolding material, as well as a platelet-rich plasma (PRP) suspension, which mimics the insoluble and soluble factors of native ECM respectively. Briefly, sodium alginate was subjected to controlled oxidation to yield alginate dialdehyde (ADA), and was mixed with gelatin and PRP in various volume ratios in the presence of borax. The formulation was systematically characterized for its gelation time, swelling, and water uptake, as well as its morphological, chemical, and rheological properties; furthermore, blood- and cytocompatibility were assessed as per ISO 10993 (International Organization for Standardization). Printability, shape fidelity, and cell-laden printing was evaluated using the RegenHU 3D Discovery bioprinter. The results indicated the successful development of ADA–gelatin–PRP based bioink for 3D bioprinting and biofabrication applications.

## 1. Introduction

With the demand for innovations and technologies to generate biomimetic organs in the field of medical technology comes the need for generation of novel three-dimensional (3D) objects that can change the face of medical science. One such innovation that has found an eminent place in tissue engineering and regenerative medicine is the 3D bioprinting technology [1]. As the name suggests, 3D bioprinting is an additive manufacturing process that uses a 3D bioprinter and biocompatible biomaterials to generate 3D tissues through layer-by-layer extrusion [2]. The resultant tissues can be used to replace, repair, or reconstruct damaged tissue/organ in the human body and fabricate 3D tissues for in vitro toxicological testing applications. Such material that incorporates cells and existing hydrogel biomaterial components to fabricate scaffolds for 3D bioprinting application is called “bioink”. Bioinks are generated from biocompatible polymers that can be tuned for their printability, biodegradability, and better mechanical property. They are physical scaffolds to which the cell attaches and proliferates to form a tissue construct [3]. Many commonly available bioinks that have been used for 3D bioprinting include collagen, alginate, gelatin, chitosan, and tissue-specific decellularized extracellular matrix. They are used either alone or in different combinations to improve overall performance in terms of cell proliferation, metabolic activity, and tissue-specific functions [4]. There have been approaches using extracellular matrices and biological components, including growth factors to develop bioinks that can support cellular growth and can be used for various tissue engineering approaches [5,6]; however, poor mechanical properties and printability limit their uses [7].

Alginate is a widely used biopolymer for the generation of scaffolds for tissue engineering applications, due to its availability, low cost, biocompatibility, and one-step gelation process [5]. Hydrogels based on an oxidized form of alginate (ADA: alginate dialdehyde) offer more reactive groups compared to native alginate, and thus were explored in combination with other polymers in a variety of cell and tissue engineering applications [6]. One of the widely used polymers in combination with ADA is gelatin (Gel), which is a thermoresponsive biopolymer derived from collagen. Gelatin is a biocompatible, bioresorbable biopolymer rich in arginine, glycine, and aspartic acid (RGD) motifs that help in cell attachment, and is therefore widely explored as a scaffolding biomaterial in tissue engineering [7]. Although the gelatin component in ADA–Gel offers cell adhesion motifs, it does not provide any other bioactive cues. One of the attractive sources of bioactive cues is platelet-rich plasma (PRP), which is enriched by a range of plasma proteins and growth factors, the most prominent being the platelet-derived growth factor, transforming growth factor, vascular endothelial growth factor, epidermal growth factor, insulin-like growth factor, and fibroblast growth factor [8]. Numerous growth factors, cytokines, and thrombin–fibrin in PRP are capable of enhancing angiogenesis, stem cell recruitment, and tissue regeneration of bone, tendon, skin, and cartilage, including cell proliferation, differentiation, and improved synthesis of the extracellular matrix. PRP has been successfully used as a therapeutic agent in the field of dermatology—for instance, in wound healing and cosmetic medicine [9]. PRP has been explored for various tissue engineering applications, as a culture supplement for enhancing cell proliferation or in tissue regeneration therapy, such as in orthopedic applications [10]. Another advantage of PRP is the autologous nature, which makes it an inexpensive and immunologically safe source in different tissue engineering applications. Its properties of enhancing angiogenesis, stem cell recruitment, and tissue regeneration are now being explored in regard to generating biocompatible bioink for cell proliferation and development [10,11,12,13].

In the current study, inspired by bioactive properties of PRP, we aim to prepare and characterize PRP supplemented ADA–Gel bioink formulation for potential 3D bioprinting applications. We followed previously reported protocols to synthesize ADA and an ADA–Gel conjugate, and subsequently performed systematic characterization studies. PRP from healthy volunteers was isolated and mixed with ADA–Gel to formulate ADA–Gel–PRP bioink. The resultant bioink was systemically characterized for its rheological, mechanical, chemical, and physical properties. Consequently, the feasibility of using ADA–Gel–PRP as a bioink for 3D bioprinting applications was verified by assessing its printability using a 3D bioprinter (RegenHu3D Discovery). Lastly, the cytocompatibility of the formulation was assessed by encapsulating the model cell line (L929, mouse fibroblast cell line), followed by a cell viability check by microscopy and CCK-8 (cell counting kit 8) assay.

## 2. Materials and Methods

### 2.1. Materials

Alginic acid sodium salt from brown algae was purchased from Sigma-Aldrich (Bangalore, India), gelatin was purchased from Gelita (Eberbach, Germany), sodium tetraborate (borax) was purchased from Fisher Scientific (United Kingdom), and sodium metaperiodate (EMSURE) was purchased from Merck (Mumbai, India). PRP was isolated from blood samples taken from healthy volunteers after Institutional Ethics Committee l approval (IEC number: SCT/IEC/1366/APRIL-2019), and L929 cell line was obtained from American Type Culture Collection (Manassas, VA, United States). All cell culture related reagents and consumables were obtained from Thermo-Scientific (Bangalore, India).

### 2.2. Preparation of ADA–Gel–PRP Bioink Formulation

Alginate di-aldehyde was prepared by controlled oxidation of sodium alginate by metaperiodate in the ethanol–water mixture, as per the earlier method described by Balakrishnan et al. [14]. Gelatin was used as received without any processing or modification. To prepare PRP, stored/fresh blood samples collected from healthy human volunteers were subjected to centrifugation at 750× *g* for 5 min. The optimization of bioink formulation was done by varying ratios of ADA and gelatin. Typically, 12% (*w*/*v*) gelatin solution was prepared in DMEM-F12 (Dulbecco’s Modified Eagle Medium/Nutrient Mixture F-12, supplemented with 10% (*v*/*v*) fetal bovine serum (FBS) and kept at 40 °C until complete dissolution. It was then mixed with a 12% (*w*/*v*) ADA solution, prepared by dissolving lyophilized ADA in 0.05 M borax. Bioink was formulated by mixing ADA/Gelatin/PRP in a ratio of 1.0:1.0:0.2 (*v*/*v*). The resulted formulation was characterized for gelation time, swelling properties, and rheological parameters.

### 2.3. Characterization of ADA–Gel–PRP Bioink Formulation

#### 2.3.1. Physico-Chemical Properties

The gelation time of ADA–Gel–PRP was determined using the tube inversion method [15]. Briefly, ADA, gelatin, and PRP solutions were mixed in a vial, incubated at room temperature, and at regular intervals, the vials were inverted to check sol-gel transition. To determine swelling index and water uptake (%), pre-weighed, freeze-dried disc samples (15 mm × 5 mm) were immersed in 2 mL phosphate-buffered saline (PBS) at 37 °C for 24 h under static conditions, and the weight change was recorded to determine swelling index and water uptake (%), as per an earlier report [16]. Samples were freeze-dried (Edwards Modulyo 4K, Pharma Bioteck, United Kingdom) at −55 °C for 12 h. Subsequently, the microstructure of freeze-dried ADA–Gel–PRP gels was investigated using a scanning electron microscope (SEM, Hitachi, Model S-2400, Tokyo, Japan) and micro-CT (Micro-computed tomography40, Scanco, Bruttisellen, Switzerland). Finally, successful completion of Schiff’s reaction and the formation of covalent bonds within ADA–Gel–PRP in presence of borax was investigated with attenuated total reflection Fourier-transform infrared spectroscopy (ATR-FTIR; 4200, JASCO FT/IR).

#### 2.3.2. Rheological Properties

Rheological property is one of the critical parameters to be considered while formulating any novel bioink for 3D bioprinting. In the current study, the rheological properties of ADA–Gel–PRP (12% *w/v* ADA in 0.05 M Borax, 12% *w/v* gelatin in DMEM-F12, PRP in a volume ratio of 1.0:1.0:0.2) were carried out by a modular compact rheometer (MCR 102, Anton Paar). ADA–Gel was also analyzed for comparison purposes. A cone plate with a cone diameter of 24 mm and a cone angle of 2.009° was used, and the measurement gap was fixed at 0.105 mm. All experiments were performed at 25 °C. The viscosity of the hydrogel was measured at a constant shear rate of 100/s. Storage modulus (G’) and loss modulus (G”) were measured at an angular frequency from 100.0 to 0.1 rads/s at an amplitude gamma of 1%.

#### 2.3.3. Biocompatibility Properties

For the hemolysis assay, the blood compatibility of the ADA–Gel and ADA–Gel–PRP hydrogels was analyzed by estimating hemolysis (%) test, as per ISO 10993-4, wherein the hydrogel discs of known size were placed in 2 mL of blood in a Petri plate. Samples were kept for agitation at 70 ± 5 rpm at 37 °C for 30 min. Subsequently, the whole blood from the sample was drawn and centrifuged for plasma separation at 1000× *g* for 15 min. From the supernatant, 100 μL of the plasma was taken and mixed with 1 mL of 0.1% (*w*/*v*) sodium bicarbonate. The absorbance of the liberated plasma hemoglobin was measured at 380 nm, 415 nm, and 450 nm in a UV-Vis spectrophotometer, and hemolysis (%) was calculated from the following Equation (1), where *free hemoglobin* is the level of hemoglobin liberated in the plasma, and the *total hemoglobin* was that from the initial whole blood count.

(1) $$\%\;Hemolysis\;=\left(\left[\frac{Free\;haemoglobin}{Total\;haemoglobin}\right]÷1000\right)\times 100$$

The cytotoxicity of the fabricated hydrogels (ADA–Gel and ADA–Gel–PRP) was evaluated using a test on extracts followed by an MTT (3-[4-C-dimethylthiazol-2-yl]-2,5-diphenyl tetrazolium bromide) assay, as per ISO 10993-5 [17]. Briefly, the hydrogel discs, of a known size having a surface area of about 1.25 cm^2^, were incubating in 1 mL culture medium at 37 °C for 24 h. After incubation, the spent culture medium containing potential leachables from the hydrogel, or otherwise termed as extracts, were collected. This was considered as 100% (*v*/*v*) extract of the test material, and was subsequently diluted with fresh culture medium to prepare 50%, 25%, and 12.5% (*v*/*v*) extracts. Ultra-high molecular weight polyethylene (UHMWPE) samples extracted in similar conditions were considered as a negative control (the one which does not harm the cells), and freshly prepared dilute phenol (1.3% *w*/*v*) in culture medium was considered as a positive control (the one that harms the cells). Different dilutions of test and control extracts were placed on a monolayer of L929 cells in a 96-well plate at 100 µL/well, and incubated in a CO_2_ incubator at 37 °C for 24 h. Subsequently, the spent medium was exchanged with freshly prepared 50 µL/well MTT reagent (1 mg/mL in medium without serum), and the cells were incubated at 37 °C for 2 h. The spent medium was discarded, and the formazan crystals were dissolved in 100 µL/well isopropanol. Cells without any treatment were considered as cell control. Absorbance was read at 570 nm in a spectrophotometer, and the metabolic activity % was calculated as per Equation (2):

(2) $$Metabolic\;activity\;\%\;=\;\frac{Absorbance\;of\;treated\;wells}{Absorbance\;of\;cell\;control}\times 100$$

### 2.4. Assessment of Printability

The printability of ADA–Gel–PRP formulation was assessed by using a state of the art bioprinting platform (Regen HU 3D Discovery). Typically, 1 mL of ADA solution (12% *w/v* in 0.05 M borax, kept at room temperature), 1 mL of gelatin solution (12% *w/v* in DMEM-F12, kept at 40 °C), and 0.2 mL of PRP (kept at room temperature) were mixed in a 35 mm culture dish to form a bioink. The bioink was then loaded into a 3 cc cartridge, as per the manufacturer guidelines. A 410 µm nozzle was attached to the cartridge tip, and it was then fixed onto print head 1 of the bioprinter. The needle height and stage were calibrated using the software, as per manufacturer instructions. A design template was prepared using the software provided with the bioprinter (size of the construct 1.5 × 1.5 cm^2^). Approximately 15 min after mixing of ADA-Gel-PRP components, the printing was initiated as per the design drawn earlier, using a pneumatically controlled extrusion print head at a feed rate of 7.5 mm/sec. The versatility of printing and shape fidelity of the construct was examined by printing multiple shapes (three shapes) and multiple layers (up to 10 layers).

### 2.5. Cell-Laden Bioprinting

A model cell line, i.e., L929 mouse fibroblast cell line, was used to assess the cell-laden bioprinting using ADA-Gel-PRP formulation. Overall, the protocol for cell-laden bioprinting was the same as described in the previous section. However, for cell-laden bioprinting, about 1 million cells in the pellet form were mixed with 0.2 mL of PRP. This was then mixed with 1 mL of ADA (12% *w/v* in 0.05 M borax) and 1 mL of gelatin (12% *w/v* in DMEM-F12) solutions to prepare the cell-laden bioink formulation. The said bioink was loaded into a sterile cartridge, and the printing was started as described in the previous section. The 3D bioprinted, cell-laden constructs, collected in 12-well plates, were fed with DMEM-F12 supplemented with FBS (10% *v*/*v*) and incubated at 37 °C for 24 h. The cell viability in the cell-laden constructs was examined by CCK-8 (cell counting kit 8) assay, as per the kit manual. Briefly, after the incubation period, the spent medium was discarded, and 500 µL of CCK-8 reagent (5% *v/v* in serum-free DMEM) was added to each well. The constructs were further incubated for 4 h in the dark at 37 °C. About 100 µL of spent medium was collected into a fresh 96-well culture plate. The absorbance of the solution was measured at 450 nm (against a reference at 650 nm), and the cell viability (%) was calculated as per Equation (2). Cell viability was further confirmed with SEM for qualitative assessment, and was also evaluated by live dead staining fluorescein diacetate (FDA) and propidium iodide (PI) using confocal microscopy.

### 2.6. Statistical Analysis

The qualitative data shown was a representative of a group of replicates (*n* = 3). The quantitative values were averaged and expressed as mean ± standard deviation (*n* = 3). Statistical significance among the test and the control values were determined by one-way ANOVA, and the values were considered significant at *p* < 0.05.

## 3. Results and Discussion

**Figure 1:**
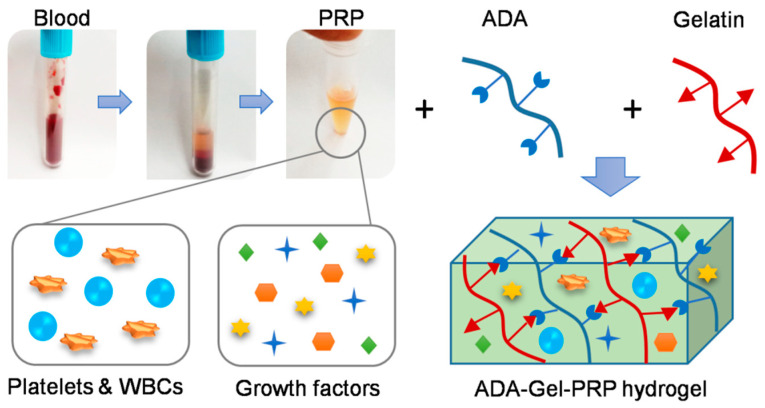
Schematic of ADA–Gel–PRP (dialdehyde–gelatin–platelet-rich plasma) hydrogel-based bioink formulation: the hydrogel network forms by covalent interaction of the aldehyde group of ADA with amine groups of gelatin through Schiff base reaction, incorporating components of PRP.

The development of tissue construction using 3D bioprinting technology has become an attractive option in the field of tissue engineering, as it offers an exciting therapeutic alternative to numerous patients. Several industries are coming forward to invest in making tissue substitutes for potential applications in the biomedical field and beyond. The bioink, a hydrogel used for 3D printing, shall meet several criteria required for an efficient tissue fabrication, such as shear thinning, mechanical properties, biodegradability, and biocompatibility. Various formulations of bioinks are being synthesized using one or more biocompatible biomaterials by following several crosslinking strategies. Here we demonstrate the formulation of ADA–Gel–PRP hydrogel and the feasibility of using it as a bioink for 3D bioprinting applications. Alginate–gelatin-based hydrogels have been used as scaffolds for cell attachment and proliferation in several studies [18]. The ADA–Gel–PRP hydrogel proposed in the current study has the inclusion of more reactive groups that enhance crosslinking and provide a better environment for cell growth. ADA is an oxidized form of alginate that has a reactive aldehyde group, which facilitates covalent crosslinking with the amine groups of gelatin and PRP through Schiff’s base reactions (Figure 1).

Gelation (gel transition) time is the time taken for a solution to become a gel. It is typically optimized to adjust for a bioink’s rheological properties. The characteristic property of hydrogel in forming a gel helps in cell encapsulation and perfects the printability of hydrogel for the generation of a 3D construct. In practical terms, we found that the mixing of ADA, gelatin, and PRP along with cells, loading this bioink into a cartridge, assembling the print head, and instrument calibration took about 3 min. Based on this, amongst various combinations and permutations of ADA, gelatin, and PRP, 12% (*w*/*v*) ADA in 0.05 M borax, 12% (*w*/*v*) gelatin in PBS, and 200 µL of PRP in a 1.0:1.0:0.2 volume ratio was found to be optimal, with a gelation time of about 4 min (rationale for the selection of optimal composition is described in Supplementary Materials). Subsequently, we investigated the swelling behavior and water uptake capacity of the hydrogel made from this optimal concentration. The swelling behavior is a critical criterion to consider, as it alters the pore volume of a hydrogel and affects the properties and performance of the gel [19]. The water uptake capacity is also an important criterion to consider, since the encapsulated cells absorb nutrients from media to maintain cell growth, mobility, and spreading. In the current study, ADA–Gel–PRP-based bioink formulation was found to have a swelling index of 0.59 ± 0.02 (or in other words, the swelling ratio of final weight/initial weight was 1.59 ± 0.02) and a water uptake capacity of ~40%, thus indicating that the hydrogel formulation does not swell much, yet holds enough media to sustain cellular activity [20].

**Figure 2:**
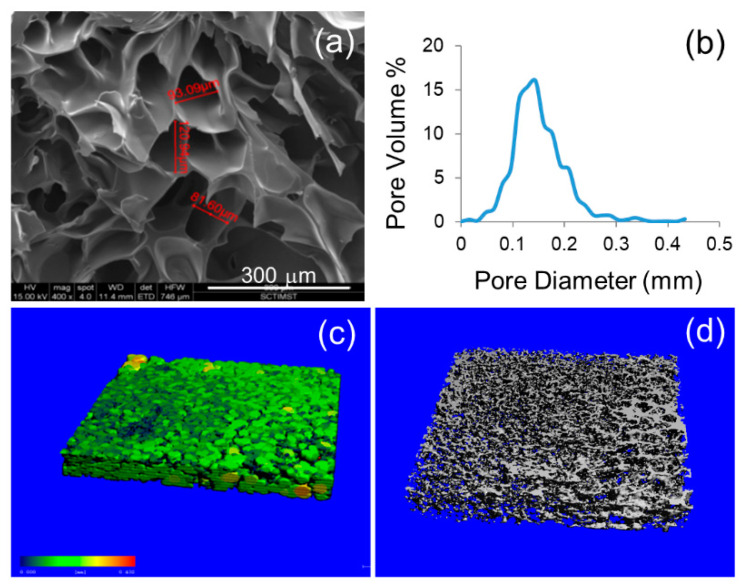
Morphological analysis of ADA–Gel–PRP hydrogel: (**a**) scanning electron microscope (SEM) and (**b**–**d**) micro-CT analysis reveal the highly interconnected porous nature of the hydrogel, with a uniform pore size and pore size distribution.

The highly interconnected porous structure is a prime requirement for any scaffold to promote proper cell seeding, attachment, and migration [21]. A considerable amount of hydrogel porosity is required for the better diffusion of nutrients and oxygen in the 3D construct, particularly in the absence of a functional vasculature system [22]. The cross-sectional morphology of the optimized hydrogel was investigated by SEM and micro-CT (Figure 2). SEM analysis showed the interconnecting porous nature of ADA–Gel–PRP hydrogels. Furthermore, micro-CT imaging was performed to analyze the pore size and porosity of hydrogel. The 3D morphology of the ADA–Gel–PRP revealed that the hydrogel was highly porous, and shows an even distribution of pores, with the pore size found to be 150 ± 50 µm and a porosity of 89% ± 5% (as analyzed through micro-CT software). However, the pore properties showed here represent the freeze-dried form of the hydrogel. They may or may not represent the wet form of the hydrogel in its absolute sense, perhaps due to the belief that the pore network would be altered upon sample swelling. Yet, since mean pore size was 150 µm in dried form, we believe that even after moderate shrinking these pores could allow efficient gas/nutrient exchange.

**Figure 3:**
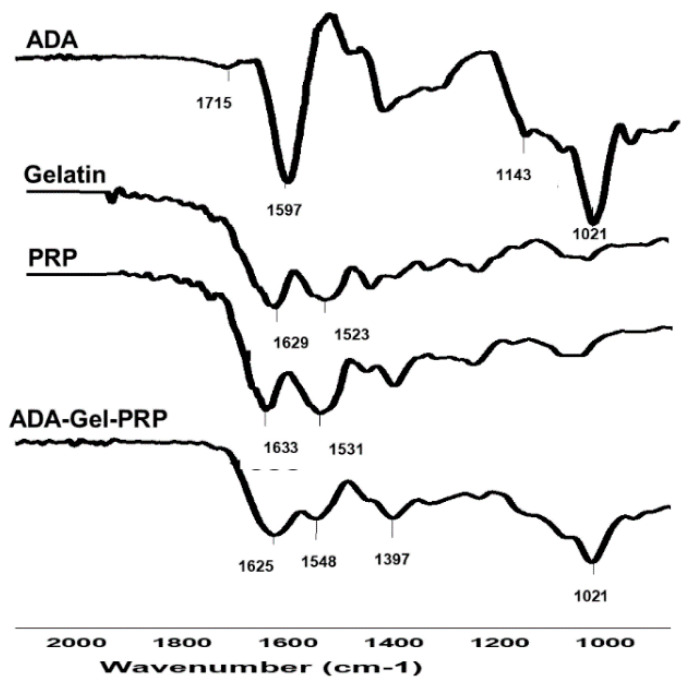
FTIR spectra of ADA–Gel–PRP formulation: a comparative observation of FTIR spectra of ADA, Gel, PRP, and ADA–Gel–PRP suggests successful Schiff’s base reactions, wherein aldehyde and amine group interaction (CH=N) was shown by broadening of the peak at 1633 and 1548 cm^−1^.

The chemical/structural features of ADA–Gel–PRP formulation and their ingredients as analyzed by FTIR are presented in Figure 3. The characteristic FTIR peaks of gelatin were seen at 1629 cm^−1^ and 1523 cm^−1^, representing C=O stretching vibration of amide I and N–H/C–N stretching vibration of amide II, respectively. Similarly, PRP was rich in proteins and exhibited similar primary and secondary amine groups at 1633 cm^−1^ and 1531 cm^−1^, respectively. ADA showed a characteristic peak at 1715 cm^−1^, representing the C=O symmetric vibration. Hydrogel formation of ADA with gelatin occurs by Schiff’s base reaction between the aldehyde groups of ADA and amine groups present in gelatin and PRP. In ADA–Gel–PRP hydrogel, the amide I band of PRP and gelatin was shifted to 1548 cm^−1^, which denotes its involvement in gelation, and thus confirms the Schiff’s base reaction within the hydrogel [23].

**Figure 4:**
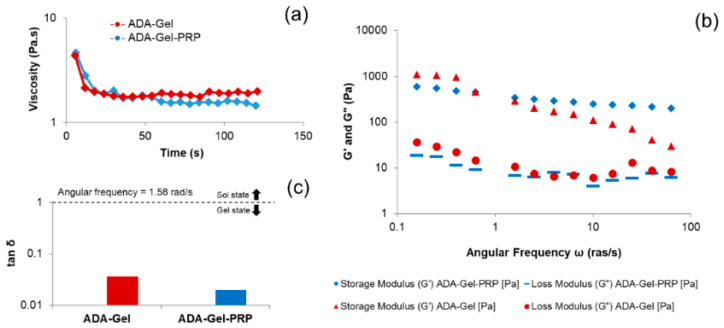
Rheological properties of ADA–Gel–PRP hydrogel: (**a**) viscosity plot, (**b**) storage and loss modulus plot, and (**c**) tan δ data show shear-thinning properties and stiffness of ADA–Gel–PRP bioink formulation.

The rheological properties of any hydrogel are critical determinants for its ability to withstand shear force while being extruded through the needle [20,24]. A material is said to be thixotropic when its viscosity decreases with time at constant shear stress [25,26]. Thus, thixotropic nature allows highly viscous hydrogels to be extruded out from the printing nozzle effectively. Consequently, the viscosity of the hydrogel was estimated with time at a constant shear rate of 100/s. Furthermore, a frequency sweep test was performed within the linear viscoelastic region of hydrogel to determine the storage and loss modulus of the hydrogel in the angular frequency range of 0.1 to 100.0 rad/s. As shown in Figure 4, the viscosity of the hydrogel decreases with time at a constant shear rate, which showed the thixotropic or shear-thinning property of ADA–Gel–PRP hydrogel. The frequency sweep test performed on ADA–Gel–PRP hydrogel exhibited a storage modulus (G’) higher than the loss modulus (G”), indicating that the elastic character was always higher when a load is applied. Furthermore, both ADA–Gel and ADA–Gel–PRP samples showed a tan δ less than 1, and thus indicated the gel-like characteristic of both these samples. However, tan δ of ADA–Gel–PRP was relatively less than ADA–Gel, and thus indicated that the presence of PRP led to stiffer gel. Perhaps the addition of PRP improves the mechanical properties, printability, and stability of hydrogel, which is an important parameter for the bioink.

**Figure 5:**
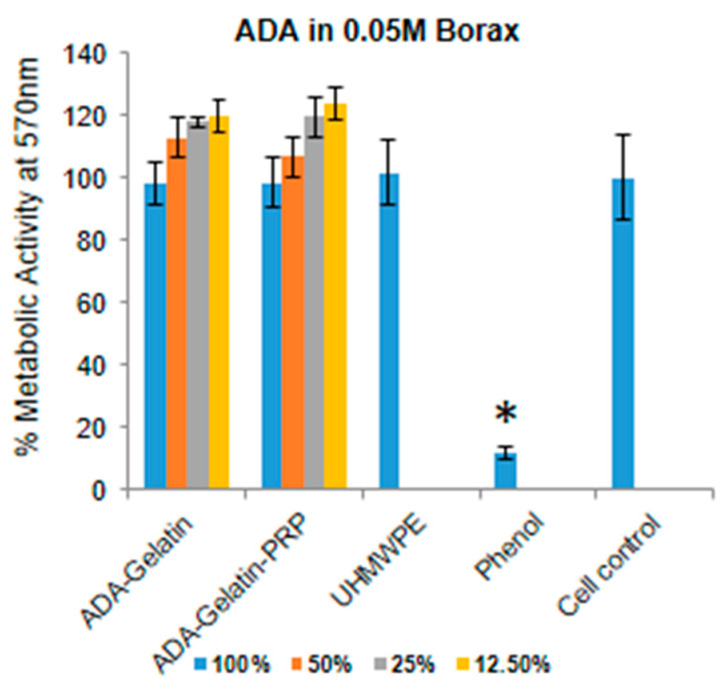
Cytotoxicity analysis of ADA–Gel–PRP bioink formulation, as per ISO 10993-5. Compared to cell control without any treatment, L929 cells treated with various dilutions of extracts of ADA–Gel and AD–Gel–PRP hydrogels exhibited ≥100% cell viability. Ultra-high molecular weight polyethylene (UHMWPE; negative cytotoxic control) showed ≥100% cell viability, and diluted phenol (positive cytotoxic control) showed <10% cell viability, as anticipated (100% extracts: undiluted culture medium with extracts of the hydrogels, at 50%, 25%, and 12.5%; *v/v* extracts: undiluted culture medium with extracts of the hydrogels mixed with fresh culture medium in 1:2, 1:4, and 1:8 *v/v* ratios, respectively). * Cell viability (%) in phenol sample was statistically significant when compared to the cell viability % in test, UHMWPE, and cell control samples, *p* < 0.05).

Hemocompatibility of a bioink is an important aspect, as 3D scaffolds materials are used to treat wounds and injuries to the patients, thus having a short- or long-term exposure of the material to the blood. Hence, ADA–Gel–PRP bioink formulation was evaluated for hemolysis as per ISO 10993-4. The presence of free hemoglobin in the plasma is caused by the lysis of red blood cells (RBCs) in the blood when in contact with the test sample. We found that ADA–gel–PRP formulation exhibited a hemolysis of 0.04%, which was found to be less than the normal range of <0.1%, as per the ISO 10993-4 standard. Besides, since 3D-printed scaffolds come into contact with the body or wound, as in the case of a skin construct, it becomes necessary to evaluate their cytotoxic nature. The key components of our bioink formulation, i.e., ADA and gelatin, were reported be non-cytotoxic and cytocompatible [14]. However, the use of borax to increase the efficiency of crosslinking may cause toxicity, depending on its concentration. In the current study, we found that ADA in 0.05 M borax mixed with Gel–PRP was found to be the optimal non-cytotoxic concentration, showing nearly 100% cell viability (Figure 5), as per ISO 10993-5 (cell viability of 60% or lower was typically considered as cytotoxic). In a recent study, Tilman et al., showed that a plasma–alginate-based bioink promotes cell growth and proliferation. Plasma contains growth factors and proteins, which helps cellular adhesion; these proteins also play an important role in cell–matrix interactions [27].

**Figure 6:**
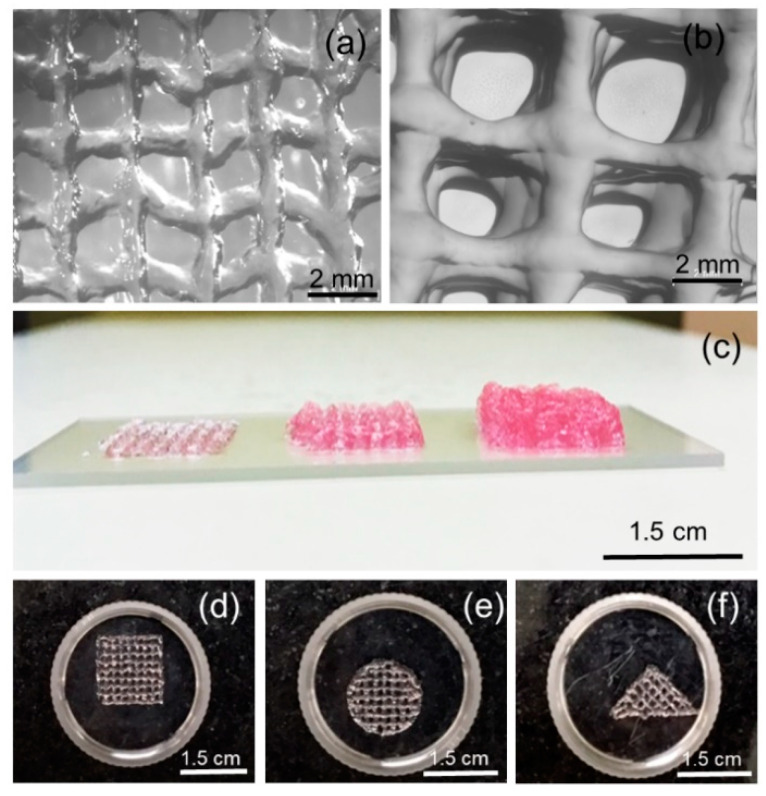
Printability aspects of ADA–Gel–PRP formulation. Stereo zoom microscope images suggested smooth and continuous filament formation in (**a**) single and (**b**) five-stack (or 10-layer) constructs. (**c**) Lateral view of single and multi-stacked constructs and (**d**–**f**) constructs in various shapes indicates the versatility of the formulation and bioprinting process.

Evaluation of bioink printability—in particular, the ability to form smooth and continuous filament and to form and sustain 3D structure—is of prime importance in bioprinting and biofabrication [28,29,30]. In the current study, we have systematically evaluated the bioprinter parameters, such as nozzle diameter, print head temperature, and feed rate, as well as the bioink formulation composition, in the process of developing a printable formulation. A formulation with 1 mL of ADA solution (12% *w/v* in 0.05 M borax, kept at ambient temperature), 1 mL of gelatin solution (12% *w/v* in DMEM-F12, kept at 40 °C), and 0.2 mL of PRP (kept at ambient temperature) were found to be yielding smooth and continuous filaments with a 410 µm nozzle and a feed rate of 7.5 mm/sec followed at ambient temperature. As shown in Figure 6, the morphological features of a single stack, as well as multiple stacks (10 layered constructs), were observed through stereo zoom microscope images. The lateral view of printed constructs having one, three, and five stacks suggests the shape fidelity of the printed constructs. Furthermore, we found that bioink formulation can be bioprinted into multiple shapes, therefore suggesting the versatility of the formulation and bioprinting process.

**Figure 7:**
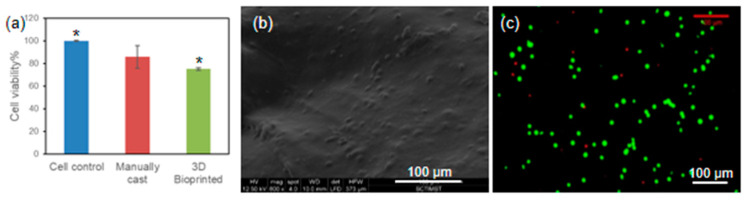
Cell viability in 3D bioprinted constructs: (**a**) CCK-8 assay, (**b**) SEM imaging, and (**c**) fluorescent live dead staining collectively suggest that the ADA–Gel–PRP formulation and the set bioprinting parameters were optimal in yielding a viable construct. * Denotes that the differences in cell viability % were statistically significant (*p* < 0.05).

It is known that the composition of any hydrogel formulation would influence cell viability, owing to the cytotoxic nature of the components involved. However, there is emerging evidence that suggests that printing parameters would also influence and seriously affect cell viability in bio-plotted constructs [31]. Therefore, in the current study, we have assessed the viability of L929 cells bioprinted with ADA–Gel–PRP formulation. After 24 h of incubation, the cell-laden constructs were subjected to a CCK-8 assay, and as evident from Figure 7a, the cell viability in 3D bioprinted constructs was nearly 80%, which was similar to the viability observed in the manually cast constructs. Perhaps, in two-dimensional (2D) culture, the cells have a treated surface with abundant cell anchoring moieties to attach, have no diffusion barrier of any kind for nutrient/gas exchange, and have abundant space to expand, whereas in 3D culture, cell viability and growth would be influenced by many factors, including but not limited to chemical composition of the ink, cell–matrix interactions, diffusion barrier to nutrient exchange, restricted freedom to expand due to gel stiffness, etc. The cell-laden construct was fixed and observed under an SEM for qualitative assessment of the cell adhesion, and as can be seen from Figure 7b, the cells were seen on/in the printed construct. The cell viability in the cell-laden construct was also evaluated by live dead staining (FDA/PI) and confocal microscopy analysis, and as presented in Figure 7c, an abundant number of viable cells (stained in green) were seen in the construct. The qualitative and quantitative analysis of cell-laden constructs confirmed that the ADA–Gel–PRP was biocompatible, and the printing parameters proposed in this study were optimal for the preparation of viable cell-laden constructs. However, the formulation composition and the printing parameters may have to be fine-tuned for specific cells and applications of interest [31].

## 4. Conclusions

Formulation of a printable bioink that has bioactive ingredients is a significant challenge, and to this end, here we report ADA–Gel–PRP-based hydrogel formulation as a novel bioink for potential bioprinting and biofabrication applications. Various combinations of ADA, Gel, and PRP in varying concentrations were studied, and a 1:1 (*v*/*v*) ratio of 12% (*w*/*v*) ADA and 12% (*w*/*v*) Gel, along with 200 µL of PRP, was found to yield a stable formulation. The gelation time was 4.0 ± 0.5 min, the swelling index was 0.59 ± 0.02, and water uptake was 37% ± 0.8%. SEM and micro-CT analysis indicated that the hydrogel has highly interconnected porous morphology with a mean pore size of 150 µm. Covalent crosslinking between ADA–Gel–PRP in the presence of borax was confirmed by FTIR spectroscopy. The rheological analysis revealed shear-thinning properties of the formulation; furthermore, it also suggested increased gel stiffness in ADA–Gel in the presence of PRP. In vitro cytotoxicity, as per ISO 10993-5, and hemolysis, as per ISO 10993-4, suggest the non-cytotoxic and non-hemolytic nature of the formulation. The printability aspect was assessed in a RegenHu 3D Discovery bioprinter, and the parameters were optimized to yield structurally stable constructs with smooth and continuous filaments. Finally, cell culture studies suggest that the cells encapsulated in the 3D bio-printed, cell-laden constructs were viable, with more than 80% cell viability. Further studies exploring this formulation in the development of multi-cellular tissue constructs are ongoing.

## Supplementary Materials

Additional methods and results are available online at https://www.mdpi.com/2306-5354/7/3/108/s1.
