# Supplementary material for: Formulation and Characterization of Alginate Dialdehyde, Gelatin, and Platelet-Rich Plasma-Based Bioink for Bioprinting Applications
Source: Bioengineering (Basel). 2020 Sep 9;7(3):108. doi: 10.3390/bioengineering7030108 (PMC7552778; doi:10.3390/bioengineering7030108)
Supplement: Supplementary file 1 [file bioengineering-07-00108-s001.zip › bioengineering-919891-supplementary.pdf]

Supplementary Information

Formulation and characterization of alginate dialdehyde, gelatin and platelet-rich plasma-based bioink for bioprinting applications

Lakshmi T. Somasekharan[1,†], Naresh Kasoju[2,†], Riya Raju[1], Anugya Bhatt[1,*]

[1] *Division of Thrombosis Research, Department of Applied Biology, Biomedical Technology Wing, Sree Chitra Tirunal Institute for Medical Sciences and Technology, Thiruvananthapuram 695012, Kerala, India*

[2] *Division of Tissue Culture, Department of Applied Biology, Biomedical Technology Wing, Sree Chitra Tirunal Institute for Medical Sciences and Technology, Thiruvananthapuram 695012, Kerala, India*

Email: anugyabhatt@sctimst.ac.in

[†]*These authors contributed equally to the work.*

1. Methods

1.1. Synthesis and characterization of ADA

The controlled oxidation of sodium alginate was carried out by the addition of metaperiodate in an equal volume of ethanol–water mixture. Typically, 20 g of sodium alginate was mixed with 13 g of metaperiodate in 200 mL of ethanol–water mixture (1:1). The reaction was carried out for 6 hours in dark at a temperature of 10 °C. The resultant solution was dialyzed against distilled water using a dialysis membrane (MWCO: 3.5 kDa, Spectrum Labs, India) for 3–4 days with periodic change of water until the dialysate was periodate-free as determined by silver nitrate assay (Sarker et al. 2014b). The dialysate was freeze-dried to make a solid form of ADA which was stored at 4 °C until further use. Subsequently, ADA was characterized by attenuated total reflectance Fourier transform infrared spectroscopy (ATR-FTIR, FT/IR-4200, JASCO) and proton nuclear magnetic resonance spectroscopy (^{1}H NMR, AMX500, Bruker).

2. Results

Table S1. Composition of ADA–Gel–PRP formulations

Formulation ID	ADA (%)	Gelatin (%)	PRP (μL)	ADA/Gelatin/PRP (Volumetric Ratio)
1	10	10	200	1:1:0.2
2	10	12	200	1:1:0.2
3	10	15	200	1:1:0.2
4	12	10	200	1:1:0.2
5	12	12	200	1:1:0.2
6	12	15	200	1:1:0.2

Table S2. Gelation time and other physical properties of the gels prepared by varying the concentration of ADA and gelatin.

Formulation	ADA Content (%, w/v, 1 mL)	Gelatin Content (%, w/v, 1 mL)	PRP (mL)	Gelation time (min)	Swelling Index	Water Uptake (%)
F1	10	10	0.2	3.1±0.5	0.70±0.06	41±2.3
F2	10	12	0.2	3.2±0.5	0.70±0.04	41±1.7
F3	10	15	0.2	2.0±0.5	NA	NA
F4	12	10	0.2	5.2±0.5	0.75±0.02	43±0.8
F5	12	12	0.2	3.5±0.5	0.66±0.03	40±1.3
F6	12	15	0.2	2.5±0.5	NA	NA
F7	12 (ADA in 0.05 M Borax)	12	0.2	4.0±0.5	0.59±0.02	37±0.8

NA = not applicable as they were eliminated due to shorter gelation time.

Table S3. Hemolysis assay results for ADA–Gel–PRP formulations

Sample	Plasma Hb	Total Hb	Hemolysis (%)
F5 AG	21.46	12.3	0.17
F5 AGP	21.56	12.0	0.18
F7 AG	5.09	13.6	0.04
F7 AGP	5.40	13.6	0.04

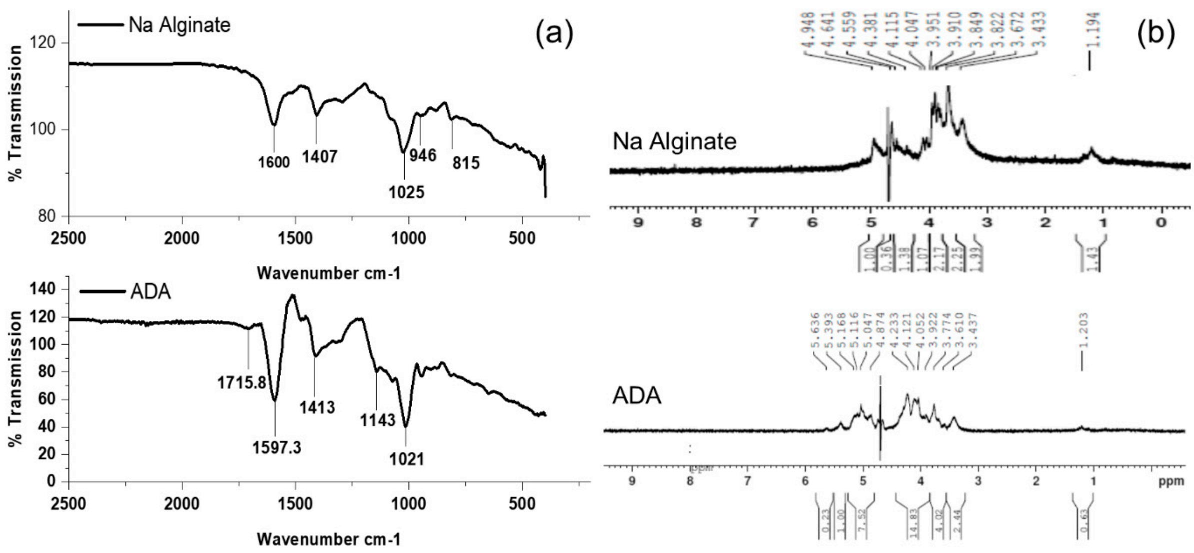

Figure S1. FTIR (a) and NMR (b) analysis of ADA.

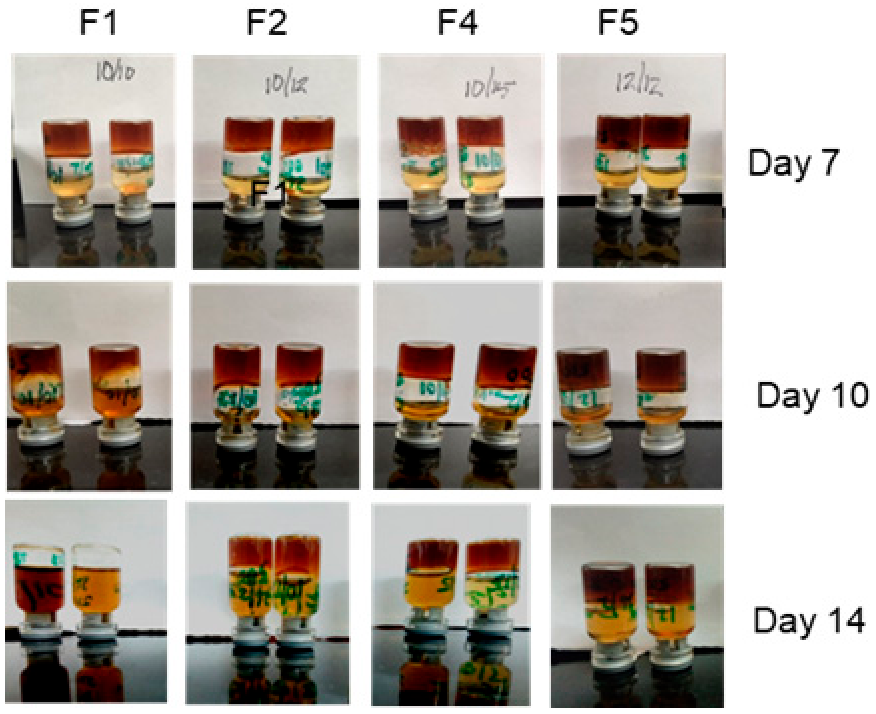

Figure S2. Degradation study of ADA–Gel–PRP formulations.

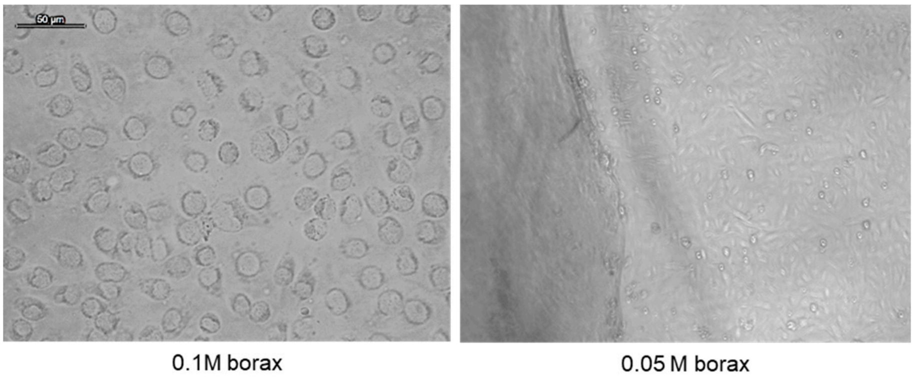

Figure S3. Direct contact-based cytotoxicity assay of ADA–Gel–PRP formulations with 0.1 and 0.05 M borax.

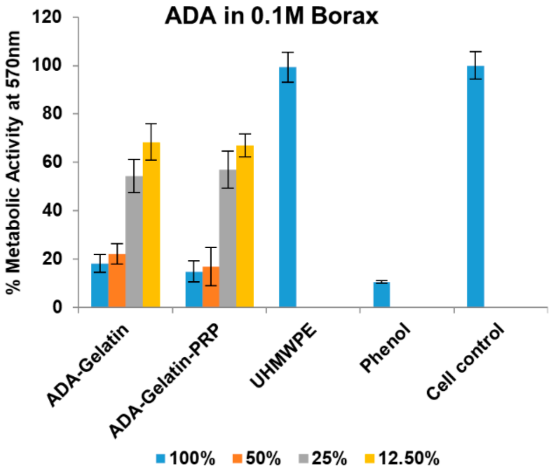

Figure S4. Cytotoxicity analysis of ADA–Gel–PRP bioink formulation with 0.1 M borax.

4. Discussion

4.1. Rationale for selecting an optimal formulation: In the current study, we have initially tried 6 formulations (labelled as F1 to F6) with varying concentrations of ADA and Gel as mentioned in Table S1. We typically mixed the components in a vial and then loaded the mixture into a printing cartridge. We found that F3 and F6 were gelling while loading the mixture into the cartridge (within 2 min) and, thus, they were eliminated for further studies.

Further, we intend to use this bioink for skin tissue engineering, which typically includes 1 week of submerged culture and 2–3 weeks of air–liquid interface culture. Keeping this in mind, we performed a biodegradation study, and as indicated in Figure S2, formulations F1, F2, and F4 were degrading relatively faster and higher than F5. We therefore eliminated F1, F2, and F4 and selected F5 for further studies. However, in the in vitro cytotoxicity studies, we found that F5 prepared using 0.1 M borax showed a cytotoxic nature, as presented in Figure S3 and S4 (as per ISO10993-5). We therefore made a new formulation, F7, wherein we took ADA, Gel, and PRP similar to that of F5, but reduced the concentration of borax from 0.1 to 0.05 M. This formulation was found to be optimal in view of the gelation time and stability in medium as well as cytocompatibility (cytotoxicity data for F7 were presented in the main manuscript).
